# Undocumented migrants’ and asylum seekers’ healthcare rights and (medical) data protection: exploring digital health opportunities and risks through the use-case of an electronic personal health record for undocumented migrants and asylum seekers in the Netherlands

**DOI:** 10.3389/fdgth.2026.1876248

**Published:** 2026-09-17

**Authors:** P. Tensen, J. M. Meulesteen, G. Trogrlić, S. A. van der Wees, E. Beune, S. J. M. van de Vijver, C. Agyemang

**Affiliations:** 1 Amsterdam Health and Technology Institute (Ahti), Amsterdam, Netherlands; 2 Department of Public and Occupational Health, Amsterdam University Medical Center (Amsterdam UMC), Amsterdam, Netherlands; 3 Institute for Information Law (IViR), Faculty of Law, University of Amsterdam, Amsterdam, Netherlands; 4 Department of General Practice, OLVG Hospital, Amsterdam, Netherlands; 5 Division of Endocrinology, Diabetes, and Metabolism, Department of Medicine, Johns Hopkins University School of Medicine, Baltimore, MD, United States

**Keywords:** asylum seekers, data protection, EPHR, healthcare access, implementation, legal frameworks, medical records, undocumented migrants

## Abstract

**Introduction:**

Despite the opportunities of electronic personal health records (EPHRs) to improve the continuity of care for undocumented migrants’ (UDMs) and asylum seekers’, implementing digital health technologies for these groups also result in legal concerns related to medical data protection. This exploratory study maps legal frameworks governing UDMs’ and asylum seekers’ access to healthcare, medical records and personal (medical) data protection in the Netherlands and examines barriers in practice according to (legal) experts. Using an EPHR as a concrete case, it further explores the opportunities, barriers, risks, and recommendations of the implementation of a digital health tool for these groups.

**Methods:**

A qualitative socio-legal study combining doctrinal legal analysis and expert interviews was applied. Thirteen (legal) experts were purposively selected and interviewed using a semi-structured guide. Afterwards, three legal experts reflected upon the results.

**Results:**

Despite the legal provisions guaranteeing UDMs’ and asylum seekers’ rights in healthcare, rights seemed inconsistently upheld in practice. Participants reported that many remain unaware of their rights, and their vulnerable circumstances could further impede the effective exercise of these rights. Participants recognised the potential of EPHRs to improve care access and efficiency, however, risks were also mentioned about (medical) data protection, particularly due to access by government agencies or other third parties, and recommended a highly secure, privacy-by-design EPHR, transparent communication, targeted education, and end-user involvement in the design.

**Discussion:**

This study highlights a central dilemma in digital health: whether and how an EPHR can enhance continuity of care for UDMs and asylum seekers without increasing risks of exclusion, forced disclosure, data misuse, or surveillance. Guaranteed software safety, transparent communication, education of users and professionals, and an inclusive design are proposed as conditions for responsible EPHR implementation, alongside stronger enforcement and awareness of existing rights.

## 1 Introduction

Digital inclusion is increasingly framed as a fundamental human right by humanitarian agencies (1). In 2023, close to 20 million forcibly displaced people lived in the European Union (EU), and close to one million non-EU citizens applied for asylum in Europe (2). Migrants and refugees experience a higher burden of various diseases due to factors such as discrimination, poor living environments, and employment insecurity, and differences in the utilisation of healthcare services compared to regular citizens (3). Specifically, UDMs have an increased risk of infectious diseases, poor dental health, mental health problems, chronic diseases, and pregnancy-related illnesses (4). Despite these healthcare needs, both UDMs and asylum seekers often face persistent barriers to accessing adequate healthcare (5–8).

Box 1 describes the legal terminology used in this paper for UDMs and asylum seekers. Supporting lists of the legal references and abbreviations used in this paper are available in Supplementary Materials 1, 2, respectively.

BOX 1 **Definition of undocumented migrants and asylum seekers.**

EU law classifies UDMs as third-country nationals staying illegally in the territory of a Member State, as set out in Article 2(1) of the 2008 Return Directive. Under Dutch law, Article 8 of the Dutch Aliens Act determines when foreign nationals enjoy lawful residence. This paper uses “undocumented migrant” to mean a ‘migrant in an irregular situation’, as used by the International Organization for Migration (IOM): “a person who moves or has moved across an international border and is not authorised to enter or to stay in a State pursuant to the law of that State and to international agreements to which that State is a party” (International Migration Law No. 34 – Glossary on Migration, 2019, p. 2). This follows migration terminology guidance discouraging criminalising or stigmatising labels and promoting rights-based terminology instead (9).

Regarding asylum seekers, Article 14 of the Universal Declaration of Human Rights states that everyone has the right to seek and enjoy asylum from persecution. An asylum seeker exercises this right by formally applying for protection in another country. The 1951 UN Convention Relating to the Status of Refugees further defines refugee status and related rights. This underpins the EU concept of ‘international protection’. This concept encompasses ‘refugee status’ and ’subsidiary protection status’, under the Qualification Directive. Together with the Asylum Procedures Directive and Reception Conditions Directive, the Qualification Directive forms the Common European Asylum System (CEAS). Under both the Asylum Procedures Directive and the Reception Conditions Directive, an ‘applicant for international protection’ is defined as a third-country national or a stateless person who has lodged an application for international protection that has not yet been subject to a final decision. Under Dutch law, the Dutch Aliens Act regulates lawful residence of foreign nationals but does not generally define ‘asylum seeker’. The applicable legal definition is therefore provided by the definition set out in the Asylum Procedures Directive.

A specific challenge for both groups that hampers the continuity of care is the limited access to health information due to missing, incomplete, or unavailable health records (5, 10, 11). This is often due to fragmented health systems that use health record systems that are neither transferable nor interconnected (12, 13). Digital health tools are frequently presented in literature as a potential solution to improve access to and quality of care (14, 15). However, in practice, individuals with precarious legal statuses, such as UDMs and asylum seekers, remain underrepresented in digital health programmes and face many hurdles using them (16).

One emerging approach that could potentially improve the continuity and quality of care for mobile groups that live in disadvantaged circumstances, including UDMs and asylum seekers, is the use of Electronic Personal Health Records (EPHRs). Evidence indicates that EPHRs for these groups have the potential to enhance data sharing, improve access to - and continuity of - care, and increase patient ownership of their medical records (10, 12, 13, 17, 18). Regardless of this potential, limited research is available on user experiences of mobile groups or legally precarious populations using EPHRs (12). EPHRs are electronic systems that allow individuals to access, manage and share their own medical information in a secure and confidential environment, that allows users to coordinate their lifelong health data and share relevant portions with those who need it, such as new healthcare providers (HCPs) (19). In this study, there is a specific focus on an EPHR that is not connected to existing HCP reporting systems.

At the same time, implementing digital health technologies results in broader ethical and legal concerns related to privacy, protection, consent and issues surrounding data ownership (17, 20, 21), for example, given that patient information is attractive to cybercriminals (22). This threat is particularly important in the context of EU borders, where ill-treatment and exploitation have been linked to power imbalances between authorities and individuals (17, 23, 24).

Also specifically regarding EPHRs for this group, concerns about data protection and security have been raised given that the fate of migrants might depend on who has access to their data (12, 13, 17, 25). For example, various HCPs in a study conducted by Tensen et al. (18) expressed concerns about potential misuse of EPHRs by, for instance, immigration authorities. Also media reports show that mobile phones can be confiscated without an individual's permission and be used for asylum purposes (26–28).

Although legal rights, such as the right to personal data protection, provide an essential foundation for the responsible implementation of digital health technologies (21), socio-legal research examining how these legal frameworks are implemented in practice, and whether vulnerable populations such as UDMs and asylum seekers can effectively exercise their rights, remains limited.

The Netherlands serves as a use-case for this exploratory study, given the previous studies done on EPHRs for mobile populations, in specific for UDMs and asylum seekers, and its relevance as a migration context due to the increasing political emphasis on strengthening and tightening asylum policies (29). An estimated 23,000 to 58,000 UDMs live in the Netherlands in 2018 (30), and 44,055 asylum seekers and so-called ‘family reunification migrants’ arrived in 2024 (31).

This study uses two complementary human rights frameworks, developed by the Office of the United Nations High Commissioner for Human Rights (OHCHR) to shape data collection and analyse results: the Human Rights-Based Approach to Data (HRBAD) and the OHCHR Human Rights Indicators Framework (32, 33). The HRBAD, developed in the context of the 2030 Sustainable Development Goals to ensure that no one is left behind, incorporates key principles, such as transparency and accountability (33). The OHCHR Human Rights Indicator Framework, in turn, provides practical tools to evaluate whether and how these principles are implemented (32).

This qualitative socio-legal study uses a novel approach in the field of digital health for UDMs and asylum seekers, combining doctrinal legal analysis with (legal) expert interviews. Using EPHRs for these groups as an use-case, this study explored the opportunities, barriers and risks from both legal and practical perspectives. The first objective was to map how the rights to healthcare access, access to medical records and data protection are defined for these groups and to explore the extent to which these are upheld in practice. These legal domains were the focus because they are directly relevant to EPHR implementation. It then examined the opportunities, barriers, risks, and recommendations associated with the responsible implementation of EPHRs for UDMs and asylum seekers. Although centred on the Dutch context, the findings may also inform the development and evaluation of digital health technologies for populations in vulnerable circumstances more broadly.

## 2 Materials and methods

The COREQ-checklist was used as a guide to report this study (see Supplementary Material 3) (34).

### 2.1 Study context

A rapid literature review shows a total of 6 EPHRs developed or used by mobile populations living in disadvantaged circumstances (12). One such initiative serves as a use-case in this study, called HealthEmove. HealthEmove is an EPHR, in the pilot phase, which allows users to store medical information and share this with (new) healthcare providers. Specifically for these target groups, this tool is not linked to existing medical data systems, given the lack of identification numbers necessary to link the tool to existing HCP software. HealthEmove is being developed by the Amsterdam Health & Technology Institute (Ahti), specifically for ‘mobile populations living in disadvantaged circumstances’ in Europe. This includes UDMs and asylum seekers who frequently change locations, though the tool does not exclude other mobile populations (18). During the course of this study, HealthEmove utilised the software of Patients Know Best, a UK-based software company and a certified Personal Health Environment (PGO) in the Netherlands and was used as an example to reflect upon by participants during the interviews. It was a web-based application that allowed both users as well as HCPs to insert medical information manually into the individual's EPHR, such as adding photos or documents or other unstructured data such as diagnosis, allergies, and medication. It was visible in HealthEmove who (HCP or user) had added which information. The EPHR could be accessed on any internet-based device; however, users often used their smartphone to enter HealthEmove (35). Data was stored on a secured Google Cloud system, hosted in Amsterdam (12). Currently, as of September 2025, HealthEmove is being redesigned separately from PKB (35).

### 2.2 Study participants and data collection

Participants were recruited through purposive sampling based on their expertise in information law, migration and refugee law, health law, data protection and information security law, social advocacy, cybersecurity, and practical experience in providing legal assistance to the target population in the Dutch and/or European context. One participant originated from the U.S., but completed a PhD in the Netherlands on human rights of UDMs in migration law, data protection law and health law. All participants were required to have substantive experience with relevant legal frameworks and/or cybersecurity; however, they did not necessarily need to hold a formal legal qualification or law degree. The snowballing technique was used to recruit participants based on referrals from participants. All participants were approached via email.

Participants were interviewed by J.M. and G.T. using a semi-structured interview guide (see Supplementary Material 4). After data analysis and drafting the results, two of the interviewed participants, one expert in health, asylum, and migration law and one expert in information law were asked via email to reflect upon the legislative frameworks and definitions. Additionally, one participant in the field of Digitalisation in Migration Law and privacy rights of migrants was asked to reflect on his input as written in the result section to make sure data was correctly interpreted. These experts provided a written reflection.

Moreover, to verify that data saturation was reached and the described legal frameworks were as complete as possible, expert validation was conducted. Using snowballing sampling technique, three additional legal experts with backgrounds in health law, EU asylum, migration and data protection law were asked to reflect on the manuscript, with a special focus on the legal frameworks. One expert in EU asylum, migration and data protection law and one expert in health law provided written feedback, and the third expert in health law provided oral feedback on the result section to P.T. and G.T. This oral feedback was recorded, with detailed notes taken during the conversation.

Before the interview the concept of an EPHR was discussed to ensure a shared understanding of the term. The interview guide was inspired by two frameworks, both developed by the OHCHR: the OHCHR Human Rights Indicator Framework and the Human Rights-Based Approach to Data (HRBAD) framework. The HRBAD framework establishes principles that legal frameworks should uphold in relation to data collection and human rights standards (33). While this framework is mainly aimed at data collection and disaggregation that respects human rights, acknowledging the inherent risks concerning the rights of data subjects, it underscores key principles also relevant for personal (medical) data access and protection: including the indicators participation, self-identification, transparency, privacy, and accountability. In the context of this study, ‘transparency’ refers to clarity and accessibility of regulations governing who can access and use medical records, while in the HRBAD framework it is mainly focussed on open dissemination of data. The OHCHR Human Rights Indicators Framework helps translate human rights principles into measurable indicators by distinguishing between structural indicators (existence of legal frameworks and institutions), process indicators (implementation and enforcement of rights) and outcome indicators (real-world effects and outcomes of rights). Structural indicators were explored through questions on the legal frameworks governing access to healthcare and health data (protection) for UDMs and asylum seekers. To address the process indicators, questions about transparency of rules and procedures and the extent to which UDMs and asylum seekers can participate in decisions regarding their health records were asked. Outcome indicators were explored by asking questions on the perceived impact of these frameworks’ risks and recommendations. Both frameworks are non-binding international guidelines, intended to guide policymakers, practitioners, and other relevant actors in implementing and monitoring human rights principles, including the collection, management, and use of data in line with international standards (32, 33). Moreover, the interview guide contained questions about participants' reflections on EPHR implementation, including on the use-case of HealthEmove. Additionally, small adjustments were made to the interview questions based on the participants' expertise. The interview questions used in the interview guide were piloted with a member of the research team and reviewed by an independent legal expert. The interview guide was prepared in both Dutch and English to support consistency across participants. See appendix II for the English interview guide.

Twenty-one individuals were invited via email or LinkedIn to participate in this study. Reasons for not participating in this study were either due to no response to (follow-up) emails or participants’ lack of expertise on the topic of this study. There was a prior working relationship with two of the participants, as they were, together with the authors of this paper, part of a ‘Digital Health for All’ research group. All interviews were conducted in Dutch. Participants could choose between meeting in person or online for the interview, and except for one, all opted to participate online via Microsoft Teams. Only the researcher(s) and the participants were present during the interview. The length of the interviews varied between 35 and 60 min. All interviews were audio recorded. Directly after recording, transcripts were transcribed verbatim by J.M., and pseudo-anonymised manually, replacing names with codes and removing identifiable details of the participant. Recordings were deleted immediately after transcription. The data is stored with Ahti in a secured organisational cloud system.

Additionally, to give an overview of the legal frameworks mentioned by the participants in the interviews, this paper used a legal doctrinal methodology to outline the existing legal frameworks relevant to the topics discussed, as well as substantiating participants’ responses by referring to the applicable legal provisions when mentioned implicitly (36).

### 2.3 Data analysis

Data analysis was informed by Clarke and Braun's iterative approach to thematic analysis (37). First, transcripts were read multiple times to become familiar with the data. An initial round of coding was conducted by J.M. Subsequently, P.T. coded several interviews to check alignment in the coding process, resulting in a revised codebook. P.T. and J.M. met regularly to discuss and align coding, and all transcripts were re-coded by both researchers using the new codebook. Both deductive and inductive coding were used, based on the applicable concepts of the OHCHR Human Rights Indicators Framework and HRBAD framework, and additionally to add new codes emerging from the data. Thereafter, codes were grouped into potential themes by examining patterns and relationships through collaborative discussions (P.T. and J.M.). As an example, codes related to “unlawfully screened mobile phones of migrants” and “systematic violations of privacy” were grouped into ‘outcome indicators - misuse of personal data’ as part of the theme privacy. Subsequently, themes were reviewed and refined by checking their coherence and distinctiveness against the data. The principles of the HRBAD framework, ‘participation', ‘privacy', ‘accountability', and ‘self-identification', supplemented by the open codes ‘Electronic Personal Health Record' and ‘legality', were used as overarching themes. Each theme was then structured using the ‘structural', ‘process', and ‘outcome’ indicators of the OHCHR Human Rights Indicator Framework and complemented by open coding. Discrepancies were resolved through discussion until consensus was reached. Themes were defined and named collaboratively, and illustrative quotes were selected to support the themes. As most legal frameworks and concepts used were in English, all data were coded in English. The software Atlas.ti was used as coding software (38).

### 2.4 Ethical considerations

Ethical approval was granted by the ethics committee of Amsterdam University Medical Center (Amsterdam UMC) (METC number 2024.1052), and approval was received on 21 November 2024. Before the interviews, all participants were sent an information letter with clear information about the study purpose, what their participation entailed, and their rights regarding confidentiality and data usage, and an informed consent form via email. All participants provided digitally signed informed consent for their participation, either before or after the interview.

## 3 Results

In total, sixteen participants were included in this study (see Table 1), of whom thirtheen were interviewed and three additional experts were consulted to read and reflect upon legal frameworks described. Participants represented a wide range of roles, including university lecturers, a policy officer, lawyers, a senior consultant in cyber security, a general practitioner, a health law expert, PhD researchers, and a legal caseworker. The participants’ areas of expertise covered all relevant legal perspectives aimed for during purposive sampling, including information law, migration and refugee law, health law, data protection and information security law, and social advocacy in the Dutch and/or European context. Some participants held expertise in particularly relevant fields such as digitalisation in migration law, or held dual professional roles, further enriching the data. This provided a broad perspective on legal, technical, and practical aspects of EPHRs for UDMs and asylum seekers.

**Table 1:** Characteristics of participants.

Participant	Role/position	Area of expertise
P1	University Lecturer, researcher, asylum lawyer and occasionally judge	Migration Law
P2	University Lecturer, lawyer	Privacy, cybersecurity, AI law
P3	University Lecturer	Asylum and Migration Law, European law, advisory boards medical research in asylum cases
P4	Policy Officer	Medical data transferability, Patient Advocacy, parliamentary group leader in Municipality for UDMs
P5	Lawyer	Social Security Law; Legal Support for UDMs, social advocacy
P6	Senior Consultant, PhD candidate	Cybersecurity, experience with digital identification for UDMs (not specifically focused on law).
P7	General Practitioner & Lecturer	Healthcare for refugees; lecturing asylum lawyers on rights regarding medical data exchange
P8	Legal advisor, Ministry advisor, Knowledge institutes	Personal health environments, health- and data protection law
P9	University Lecturer - Professor	Information Law
P10	PhD candidate	Digitalisation in Migration Law, privacy rights of migrants
P11	University Lecturer, lawyer	International Migration and Refugee Law, human rights of UDM
P12	Lawyer	Migration rights, medical grounds for asylum cases
P13	Legal Caseworker	Refugee Legal Support
Additional experts		
P14	University Lecturer - Professor	EU asylum-, migration- and data protection law
P15	University Lecturer, lawyer	Health Law
P16	University Lecturer - Professor, Director	Health Law

### 3.1 General barriers for acting upon rights

Before describing the legal frameworks relevant to EPHR implementation for both UDMs and asylum seekers in the context of this study, it is important to note a recurrent theme in the interviews. Overall, multiple participants described that for both UDMs and asylum seekers many are unaware of their (health) rights, and even if they are aware of their own rights, their vulnerable position hinders them from effectively exercising their rights. This is illustrated by P9:

“In practice, we are talking about a vulnerable group of people who are not in a very strong position and who may find themselves in situations where they are unable to properly assert or have their rights respected” - P9

Fear of acting upon one's own rights was also frequently mentioned by participants. P3 explained that many asylum seekers and UDMs come from countries where governments hold substantial power, making them hesitant to refuse authority figures.

“Resisting, saying ‘no’ to authorities; that's not something most people would dare or want to do, or people are very afraid that they will be disadvantaged. […] if you come from a country where the government simply has a lot of power; try saying no to someone in uniform” - P3

P6 added that unfamiliarity with the new country’s culture can amplify this fear, and P11 noted that UDMs may fear deportation when exercising their rights.

### 3.2 Rights to healthcare, medical records, personal data protection, and practical barriers

The following section examines the legal frameworks governing access to healthcare, medical records, and personal data protection, and subsequently describes practical barriers. Figure 1 gives an overview of the existing relevant legal frameworks mentioned by participants, which were further built upon using a legal doctrinal methodology.

**Figure 1:**
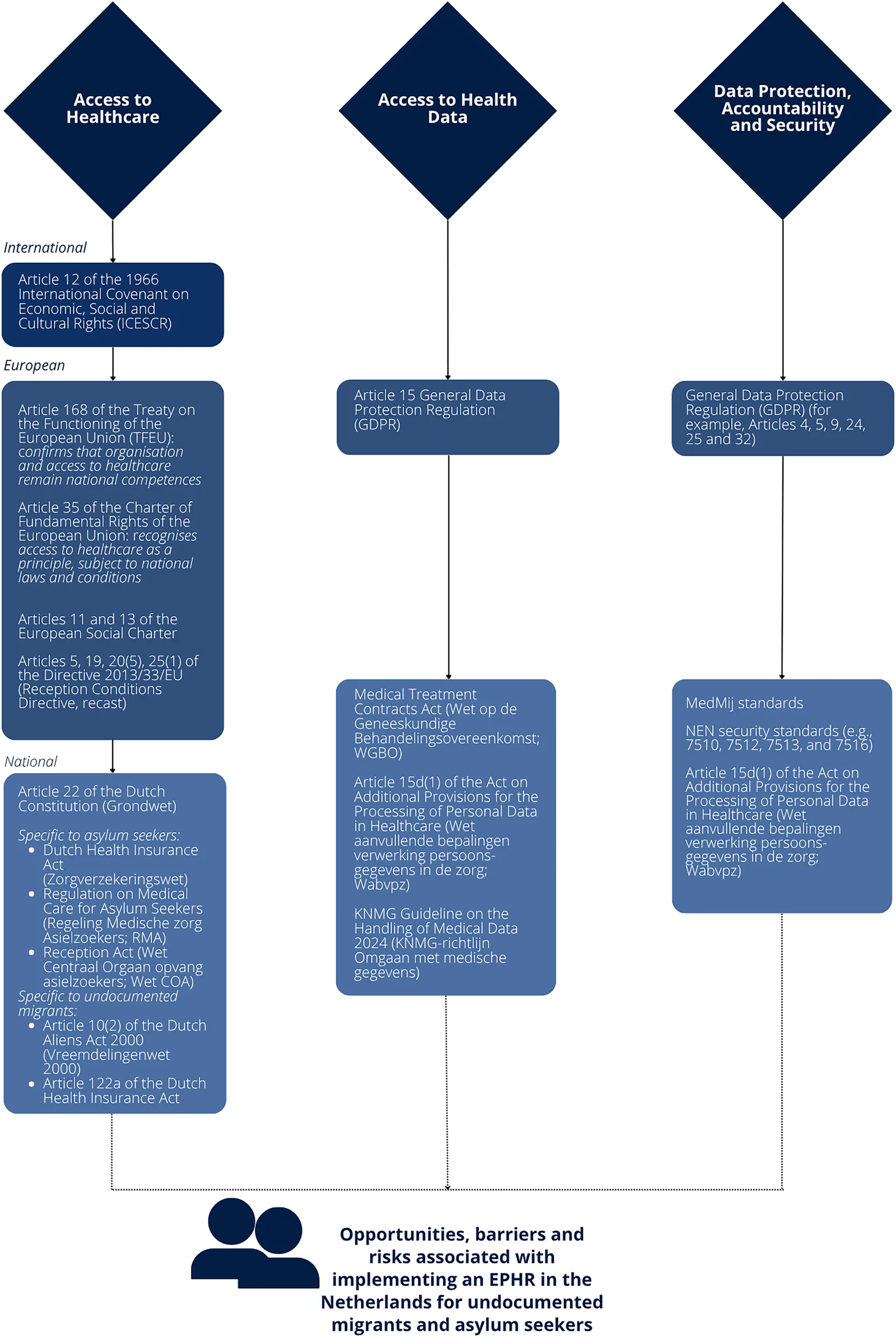
Overview of legal frameworks concerning access to care, access to health data, and data protection for UDMs and asylum seekers addressed in this paper.

#### 3.2.1 Rights to healthcare

Multiple participants underlined that everyone, regardless of legal status, has ‘the right to healthcare’ in the Netherlands and in the EU. Box 2 outlines the relevant international, EU, and (Dutch) national legal frameworks relating to the right to healthcare in the context of this study.

BOX 2 **Definitions of the right to healthcare.**

Access to healthcare is recognised in international, EU, and national legal frameworks, although their legal nature, scope and enforceability differ substantially. The right to health as a basic social right is first set out in Article 12 of the International Covenant on Economic, Social and Cultural Rights (ICESCR). Here, the UN Special Rapporteur on Health emphasised that UDMs should not be denied access to medical care (United Nations and World Health Organization, ‘The right to health’, No. 31, 2008, p. 20). Additionally, the right to health is set out in specialised UN human rights treaties, including Article 12 of the Convention on the Elimination of All Forms of Discrimination against Women (CEDAW), Article 24 of the Convention on the Rights of the Child (CRC) and Article 25 of the Convention on the Rights of Persons with Disabilities (CRPD). Under EU law, Article 168 TFEU requires a high level of human health protection to be incorporated into the definition and implementation of all Union policies and activities, while confirming that healthcare organisation and delivery, including access conditions, remain within Member State competence. Article 35 of the Charter refers to “everyone” when recognising the right of access to preventive health care and the right to benefit from medical treatment. However, this is a principle to be ensured under national law and practice, not a directly enforceable EU right. The Reception Conditions Directive (RCD) operationalises these principles within EU asylum law, obliging Member States to guarantee access to necessary healthcare for asylum seekers, who are referred to as applicants for international protection, including emergency care and essential treatment for physical and mental illnesses, see, for example, the Articles 5, 19, 20(5), and 25(1) of the RCD. Articles 11 and 13 of the European Social Charter oblige State parties to ensure adequate healthcare in cases of sickness, explicitly emphasising the duty of states to provide assistance to persons without resources. Under Dutch law, Article 22 of the Dutch Constitution states that the government must take measures to promote public health, which is implemented through legislation adopted under Article 22(1).

In the Netherlands, UDMs are entitled to ‘medically necessary care’, grounded in Article 10(2) of the Dutch Aliens Act, which exempts such care from the general exclusion of provisions for people without lawful residence. Despite not being able to obtain health insurance under the Dutch Health Insurance Act, the right to such care is set out in Article 122a of that Act, which allows HCPs to seek reimbursement for care provided to undocumented migrants who are unable to obtain insurance, provided they have first attempted to recover the costs from the patient themselves. In order to clarify the concept of ‘medically necessary care’, the Klazinga Committee defined it in 2007 in what is now recognised as a guideline.^1^ It refers to ‘responsible and appropriate medical care’, meaning “care is effective and targeted, and is given in a patient-oriented manner and is fine-tuned to the patient's actual needs. In doing so, the doctor bases the indication on a thorough analysis of the patient's health problems and indicates in accordance with the standards of the profession and in a ‘cost-effective manner’” (Commissie Medische zorg voor (dreigend) uitgeprocedeerde asielzoekers en illegale vreemdelingen, 2007). The Stichting Klachten en Geschillen Zorgverzekeringen (SKGZ), an independent complaints and expert centre for health insurance, plays a key role in providing Dutch healthcare providers with information on reimbursement for care provided to uninsured patients through guidance documents (Wegwijzer onverzekerden, SKGZ).

Under national law, asylum seekers in the Netherlands are entitled to reception, including necessary medical care, under the Dutch Asylum Seekers Provisions Regulation. The Centraal Orgaan opvang Asielzoekers (COA) organises and funds healthcare access for this specific group (COA, n.d.-a) under the COA Act. Rejected asylum seekers are entitled to reception during the statutory 28-day departure period following the rejection of their application (COA, n.d.-a).

#### 3.2.2 Rights to access to personal health data and practical barriers

Participants explained that patients, including both UDMs and asylum seekers, have the right to access their personal (health) data and request a copy of information relating to their treatment, which is important for EPHR implementation. Box 3 provides an overview of the EU and Dutch legal frameworks structuring the right of access to personal health data.

BOX 3 **Right to access personal health data.**

At EU level, the General Data Protection Regulation (GDPR) forms the overarching legal framework, providing a right of access to personal data, including medical data, under Article 15 GDPR. In the Netherlands, rights relating to medical files and health data are further regulated by the Medical Treatment Contracts Act (WGBO). Building on the WGBO, Article 15d of the Act on Additional Provisions for the Processing of Personal Data in Healthcare (Wabvpz) further provides that patients may access their patient file electronically and receive a free copy of their electronic medical record, including a logging overview detailing who has accessed their health record, as set out in Article 15e Wabvpz. Health law is also characterised by guidelines developed by the medical profession, including the 2024 KNMG guideline on Medical Data Management.

UDMs and asylum seekers are considered ‘data subjects’ under Article 4(1) GDPR. The absence of lawful residence status should not affect the applicability of the abovementioned provisions, including rights relating to access to medical records, electronic records, and logging information, where applicable, nor the right to obtain copies of these records.

Moreover, data subjects may exercise the right of access to medical records through an authorised legal representative under Articles 7:465 and 7:456 of the Dutch Civil Code. However, this does not transfer the right itself, which remains with the data subject.

Participants explained that both UDMs and asylum seekers often do not currently have access to their medical records and do not receive adequate information on how to obtain them.

Participants noted that for UDMs, accessing health records is considered difficult in practice. Even when UDMs try to obtain their own records, barriers are mentioned, such as financial constraints, travel costs, or limited ability to act independently due to systemic obstacles. P13, a legal case worker, explained that organisations who support UDMs also experience barriers as legal representatives to access medical records for their clients.

“GZA (in the Netherlands, the GZA is contracted to provide care during asylum) is also very difficult to deal with, because they do not want to share this with [organisation name]; they only want to share it with lawyers or sometimes only directly with the client themselves” - P13

Furthermore, she explained that her clients would rather obtain their health records in person than requesting them online, even though this is often not feasible:

“For the other client, it's really an insurmountable task they can't do [requesting medical records]. Even just due to travel costs, for example.” - P13

Moreover, P5 emphasised that the situation of many of his UDM clients is so precarious in the Netherlands that “*UDMs can be grateful if anything is recorded in the registration systems of healthcare providers at all*”.

For asylum seekers, participants explained that lawyers often request medical data on behalf of their clients when they believe it could influence the outcome of the asylum decision. P12 stressed that their clients often do not receive any information about the procedure of requesting medical data:

“Most clients don't even understand that they need to give me permission before I can request their data. So, whether it's really explained to them in a way they can understand - no, I doubt that” - P12

While requesting medical records for asylum seekers by legal representatives from the GZA is generally well-organised according to several participants, participants noted that lawyers face more barriers with other HCPs, such as general practitioners or hospitals, who sometimes view providing medical records as too complicated or time-consuming. P7, a medical doctor advising lawyers on health and asylum law, adds that for HCPs, unfamiliarity with legal frameworks or overly strict interpretation of privacy rules often leads providers to wrongly assume that they cannot share information with lawyers, even when legally permitted. Additionally, some providers request payment for releasing records, as P7 illustrated:

“I always advise lawyers to say: ‘The patient has no money, so please do not charge any fees.’ Patients have the right to access their own records without paying” - P7

#### 3.2.3 Personal data protection and forced access

Participants referred to the GDPR as the overarching European framework to safeguard medical data and privacy rights of individuals in the Netherlands. At the national level, participants referred to the WGBO and the Individual Healthcare Professions Act (in Dutch: Wet BIG, concerning medical professional confidentiality). Additionally, the MedMij Agreement Framework, including the NEN standards^2^, sets out comprehensive rules for secure exchange of health data in the Netherlands. While Box 3 described the GDPR in the context of *access* to personal health data, Box 4 turns to the GDPR in relation to data *protection* more broadly.

BOX 4 **Description of protection of personal data in GDPR.**

For privacy and data protection, the General Data Protection Regulation (GDPR) sits within a broader legal framework that includes the European Convention on Human Rights (ECHR) and the Charter. The GDPR codifies the principles set out in Articles 7 and 8 of the Charter and constitutes the binding European legal framework for data protection. It defines personal data as information relating to an ‘identified or identifiable’ person (Article 4(1), Recital 26 GDPR), which makes the question of when a person is considered ‘identifiable’ particularly important in this context. Furthermore, once it has been established that personal data are involved, the concept of ‘processing’ becomes relevant (Article 4(2) GDPR). In this context, ‘processing’ encompasses almost every action involving personal data, including, but not limited to, storing, analysing, distributing, copying, retrieving, anonymising, aggregating, and destroying data. Additionally, if personal data relating to a person is being processed, that person is referred to as the ‘data subject’ (Article 4(1) GDPR).

Participants explained that medical data is considered a ‘special category of personal data’, which means that such data generally is not allowed to be processed, except under ‘specific circumstances’ such as national security and counterterrorism, or situations in which explicit consent is given by the data subject (Art. 9(2)(a) GDPR). Additionally, P10 explained the importance of the ‘purpose limitation principle’ under the GDPR Article 5(b), which requires data to be collected for specified, legitimate purposes.

Although most participants agreed that the protection of personal data provided by the GDPR would, in theory be sufficient for both UDMs and asylum seekers, in practice, it is not always experienced as sufficient. This is further reported by P2, who believed that the GDPR itself offers adequate protection for these groups in the Netherlands, but enforcement is lacking:

“The GDPR has all kinds of good rules. If companies, organizations, and governments complied with them, I think we would already be much better protected. […] It is not monitored very strictly” *- P2*

Contrary, P8 noted that according to him the GDPR is not sufficient to protect individuals, given that people are not always sufficiently informed to provide informed consent, given that some people are living in vulnerable circumstances, experiencing financial or psychosocial stress, and that there is no clear legal basis against trading personal health data.

A specific example reported by participants of a lack of GDPR enforcement, monitoring and a lack of respect for privacy rights of individuals, is ‘phone screening’ for purposes outside verification of someone's identity. With respect to the purpose limitation principle, P10 explained in a follow-up email after the interview that mobile phones of migrants are allowed to be screened, so-called ‘smartphone screening’, by the Department of Immigration Police, Identification and Human Trafficking and the Royal Netherlands Marechaussee (In Dutch: Afdeling Vreemdelingenpolitie, Identificatie en Mensenhandel (AVIM) and the Koninklijke Marechaussee (KMar)) for the specific purpose of identity verification. However, it is noted by multiple participants that smartphones can also undergo examination for information relating to travel routes and indications of human trafficking, smuggling or terrorism.

P10 reported that such data may be valuable to other (immigration) authorities and could be repurposed for objectives beyond their original intent. P10 noted that for example, in cases where the information on the device contradicts the asylum seeker's reported details, it potentially becomes relevant for the IND, the authority that assesses the asylum application in the Netherlands. If certain data indicates human smuggling or terrorism, this may be of interest to authorities such as the General Intelligence and Security Service (in Dutch: Algemene Inlichtingen- en Veiligheidsdienst (AIVD)). As such, P10 raised questions about whether such further processing complies with the GDPR and the purpose limitation principle.

“Then data is being forwarded to other authorities and processed for purposes other than those it was originally collected for. The question is, is that AVG (GDPR) compliant?” - P10 (cited from follow up email after interview on 2025-11-11)

This latter concern is shared by P3 and P14. P3 explained that several court rulings found that mobile phones have been extracted without a sufficiently clear and precise legal basis (dates of rulings specified by P10 on 2024-04-03 and on 2025-01-22). P10 further reported that, although the practice of phone screening is currently grounded in Article 59(8) of the Dutch Aliens Act 2000 (Vreemdelingenwet 2000), the Administrative Jurisdiction Division of the Council of State has ruled that this provision does not meet the clarity and precision requirements set by the GDPR and the European Court of Human Rights (ECHR).

P3 described cases that phones are sometimes confiscated when people are taken into custody for example if they are no longer legally permitted to live in the Netherlands, and accessed via facial recognition or under pressure, which is not legally permitted, as illustrated by P3:

“That could be when someone is taken into custody because they no longer have legal residence in the Netherlands, and then their phone is confiscated. And what they do then is simply use facial recognition. And then you have access to that phone. […] Or look, if they can't do that immediately, they could also pressure people to show certain data, and that's not allowed, but it happens anyway” - P3

When it comes to violations of privacy, P12 reported that infringements made by authorities are not always actively sanctioned, for example, because asylum seekers themselves are not aware of the violation. Even when legal violations are recognised, participants explained that asylum lawyers are often unable to assist effectively because their expertise is focused on the asylum procedure, and high workloads leave little time to address other legal issues. P3 mentioned that violations often go unnoticed unless someone actively raises the issue:

“The lawyer only finds out that this happened when someone is, well, outraged or concerned about it.” - P3

### 3.3 EPHR implementation: opportunities, barriers, risks and recommendations

Many participants considered the concept of EPHR implementation for these groups very useful. Nonetheless, specific barriers and (legal) risks were raised mainly regarding privacy and data protection.

Table 2 shows an overview of the opportunities, barriers, risks and recommendations as mentioned by the participants, described below.

**Table 2:** Overview of opportunities, barriers, risks and recommendations of an EPHR for UDMs and asylum seekers.

Opportunities of an EPHR	Barriers of an EPHR	Risks of an EPHR	Recommendations
Improve healthcare access	Difficulty gaining some individuals' trust in an EPHR	Medical data can fall into the ‘wrong’ hands: • Being pressured by authorities to provide access to health data • Technical access through data hacks • User provides access to medical data ‘too easily’	A secure digital environment: privacy-by-design
Improve healthcare efficiency	Challenging to reach and guide individuals with low literacy, mild intellectual disability or low digital skills	Result of risk: Medical data could potentially be used against an individual in asylum decisions	Be transparent about functionalities and risks of an EPHR
Empower individuals with control over their own personal medical data: *informational self-determination*		Result of risk: Large-scale data collection of migrants could eventually be used to train automated decision-making systems	Educate on functionalities of an EPHR and on people's rights
Information in EPHR could serve as proof in asylum decisions		Risk of a lack of data authenticity and falsification of medical data in an EPHR which could interfere with clinical decision making	Make an inclusive platform and involve end-users in the design and implementation

#### 3.3.1 Opportunities and barriers of giving individuals access to an EPHR

Participants reported an EPHR as a way to improve healthcare access and system efficiency, and to empower both UDMs and asylum seekers by giving them control over who accesses their health information and under what circumstances. P3 described this as regaining a sense of control, provided the EPHR is well organised:

“It can lead to a certain level of empowerment, meaning that as an individual you regain a sense of control over your own situation […] It is really important that this is well organised for people. I truly see the benefits of it; it is a very good idea that you can have access to it yourself ” *- P3*

P8 and P10 described this autonomy as *informational self-determination*, while P9 clarified it reflects meaningful control over data sharing rather than full legal ownership.

For asylum seekers, some participants also noted that an EPHR could support asylum procedures by documenting evidence of torture, trauma, or female genital mutilation, and P3 mentioned it might even support a postponement-of-departure request under Article 64 of the Dutch Aliens Act 2000 (in Dutch: Vreemdelingenwet 2000). However, P15 was sceptical this would meaningfully affect asylum outcomes, since such evidence is still subject to credibility assessment:

“Even if you have that [foreign documentation of a declaration of torture], it is questioned here in the Netherlands, and it still comes down to an assessment of credibility. The IND has doctors who will examine, ‘is that really correct?’” - P15

At the same time, several participants stressed that an EPHR is a practical tool, not a structural fix to solve the problem of restricted access to healthcare.

Barriers to EPHR implementation were also raised by participants. First, difficulties of building trust in EPHR systems among both UDMs and asylum seekers, particularly given that some individuals may be traumatised and distrustful of governmental agencies, as illustrated by P10:

“How do you gain the trust of people who are traumatised and have been on the road for a long time and who have to deal with all kinds of government agencies […] that is quite complicated.” - P10

Second, how to reach and guide individuals with low literacy, mild intellectual disability, low digital skills or a lack of digital means, who will need the most guidance with an EPHR.

“People are often simply unreachable, […], because their phone credit has run out or their phone battery is dead and they can't charge it — all these small, frustrating practical issues.” - P7

Finally, the vulnerability of both groups was further underscored, referring to the difficulties of acting upon their rights in practice.

Barriers specific to HealthEmove were also reported. P4 noted its lack of integration with existing HCP information systems, P7 stressed the difficulty providers may face locating relevant information when many documents are uploaded, and P9 noted the dependency of HealthEmove on an external software party.

#### 3.3.2 Risks of EPHR implementation

While many participants emphasised opportunities offered by an EPHR and raised some practical barriers to its implementation, other participants focussed on the risks associated with its implementation for UDMs and asylum seekers.

##### 3.3.2.1 Medical data falling into the hands of authorities or third parties

A potential risk reported by participants, related to EPHRs for UDMs and asylum seekers, is that medical information could fall ‘into the wrong hands’, referring to unauthorised access by authorities or other unspecified third parties.

Multiple sources of this risk were mentioned. The first source of unauthorised access was that individuals could be pressured by institutional/state access. Multiple participants noted that authorities may pressure individuals to grant access to, for instance, their mobile phones and similar devices. This concern is particularly relevant regarding access to asylum seekers’ devices by state authorities, with or without consent, and is linked to the phone screening practices described in 3.2.3.

The second source is technical access, referring to unauthorized access via hacking EPHRs. Several participants argued that sufficiently motivated third parties, including state actors or ‘malicious actors’, could potentially access EPHR data regardless of security measures in place. This risk is compounded for UDMs without an identification number, as identification numbers are typically used as anchors in secure digital authentication.

Some comments regarding risks were specifically related to HealthEmove, which stored data in a secured Google Cloud environment. While P6 and P8 considered Google Cloud secure enough at present, they emphasised that safeguards should be continuously updated as new risks emerge. Similarly, P9 raised concerns about Google's US jurisdiction.

The third source is access to medical data provided by the users themselves. Participants highlighted that UDMs or asylum seekers may share their medical data too easily, without fully understanding what they are disclosing, to whom, or the potential consequences, increasing the risk of misuse of their information.

“*On the one hand it is very useful that they can share it [their record] themselves. On the other hand, it poses a risk that they share it too easily, that information can fall into the wrong hands” - P13*

Also, P8 explained that doctors are protected against requests of medical data sharing of their patients under the WGBO and the Individual Healthcare Professions Act (in Dutch: Wet BIG), however, individuals particularly UDMs and asylum seekers, do not have the same legal protection outside the medical context, and therefore wondered “*how free are you to give consent?*” additionally, P10 raised that the dependent position of UDMs and asylum seekers in relation to authorities undermines the voluntariness required for valid consent of sharing personal information.

“Migrants arriving here are in a dependent position relative to the government. So, you are actually very dependent on the government to obtain status here. Therefore, there is no such thing as freely giving consent in that context.” - P10

P14 reflected on this and shared these concerns, and noted particularly the vulnerable position of children, both in relation to data protection and the requirement of prior informed consent.

This next concern links to the responsibilities of the end-user. P7 pointed out that the system could be safe, but the individual also needs to be able to use it safely in practice themselves. For instance, risks were believed to arise when individuals temporarily store photos of medical documents on personal devices instead of within the secured EPHR environment. It was raised that there is a trade-off between the strong security of an EPHR and the usability of a tool.

“You can't fix the user. Users don't read instructions, users aren't concerned with data sharing at all” - P6

Some participants noted that, if medical information were to be found on phones, it could potentially be used for migration purposes for both groups. P10 explained that medical data obtained from phones used for identification purposes, could in the future also be used to assess the credibility of an asylum claim, referring to current political debates advocating for the use of such data in asylum procedures.

“So, I can imagine that if, say, a migrant says, ‘I was tortured in my home country’ — I'm just throwing that out there — a medical file could maybe be used to say, ‘well look, your records don't show that at all.’ Or that they don't reflect that picture at all” - P10

While, being sceptical that data could be used against an individual, P15 did explain that if for example an asylum seeker's medical data indicates that they had been in safe EU countries prior to their arrival in the country where they apply for asylum, the individual may, among other consequences, be transferred back to the first EU country of entry due to the Dublin Regulation (EU) No. 604/2013 (Dublin III).

However, both P3, P12 and P15 argued that the likelihood of these situations is relatively low. Additionally, multiple other participants mentioned that they do not yet see how medical data could negatively influence asylum decisions for asylum seekers.

“I don't immediately see a risk that it could be used against them, for example, from the perspective of the asylum procedure. I can more easily imagine that someone would over-share information, […] Usually, that does not cause much harm, apart from the privacy aspect” - P13

Concerning UDMs, P11 and P15 expressed scepticism about the claim that an EPHR would introduce additional privacy-related risks. P15 argued that the possibility of deportation is not a consequence of carrying an EPHR, but rather stems from holding a precarious legal status that may result in immigration detention and/or deportation.

Another potential harm mentioned by participants associated with unauthorised access to data is the potential of building a central database of UDMs and asylum seekers. P10, who further warned that if governments gain access to data of migrants, noted that large-scale data collection of asylum seekers could potentially be used in the future to train automated decision-making systems for asylum decisions, “*the more information is accessible to the government, the more risks this poses on individuals*”:

“For example, you may see that people from Syria often refer to the risk of being tortured in their country of origin. However, medical records may not show that this is actually the case in many instances. This could potentially contribute to the development of biases within government authorities, leading them to perceive these individuals as less credible when they make such claims” – P10

P2 reflected that this concern is particularly relevant if an EPHR uses a centralised database rather than having the ‘key’ with the user.

One way to mitigate state access was mentioned by P10, who emphasised that current legislation on smartphone screening needs to be specified. The legislation should clearly define the conditions under which phones can be accessed, what data may be collected, how long it can be retained, and which officials are authorised to access it.

##### 3.3.2.2 Risk of a lack of data authenticity and falsification of medical data

Another risk identified by participants concerned the ‘authenticity of user-uploaded data’. This risk stems from the EPHR system itself, for instance HealthEmove that allows users to upload their own information. Regarding the severity of this risk, some participants raised concerns that with a lack of digital identity, in the case of UDMs, and authentication mechanisms, EPHRs could allow for fraudulent or unauthenticated data uploads. This could potentially create the risk that unverifiable or even falsified medical letters could be entered in a patient's record and be relied upon in clinical decision-making. As one participant illustrated:

“What authenticity features does this have? How can you be sure that it really comes from a general practitioner and not from a friend in the attic who said: Yes, no, this patient really needs oxytocin, because he has a prescription for it.” - P6

Participants did not report a known instance of this occurring; however, it was mentioned as a potential gap in the EPHR system.

A way to mitigate this risk was reported by P6, who explained that HCPs must critically assess the authenticity of such documents before relying on them for clinical decisions.

#### 3.3.3 Recommendations for EPHR development

To further mitigate risks and barriers as discussed above several recommendations were provided by participants.

##### 3.3.3.1 A secure digital environment: privacy-by-design

Given the risks of unauthorised access and misuse by authorities or other unspecified third parties, participants emphasised the need for a secure digital environment that prevents unauthorised access. Several referred to privacy-by-design (Article 25 GDPR) as a guiding principle.

Recommended safeguards included two-factor authentication, encryption, secure closed cloud environments with restricted access, and data minimisation to ensure that only necessary information is collected in the EPHR.

“I would strongly recommend developing a secure cloud environment that cannot be accessed by law enforcement authorities, such as the Aliens Police or the Royal Netherlands Marechaussee. This would enable data to be removed from the phone immediately after photographs are taken”- P10

Participants also stressed that users should retain exclusive control over access to their data, with P2 arguing that encryption keys should remain solely with the user:

“Yes, I think that all centralised solutions where the key does not lie with the user — let's say, the undocumented — could in theory be abused by the government, which could indeed be a major problem, especially for the undocumented.” - P2

A recommendation mentioned for HealthEmove specifically, was moving away from US data clouds to a European cloud as a potential future solution. While the current setup of HealthEmove was considered sufficiently secure, several participants stressed the importance of continuous improvement: “*I think that under the current circumstances, you can't do much better, but it really should improve as soon as possible.” - P8.*

##### 3.3.3.2 Be transparent about the functionalities, risks and responsibilities of an EPHR

Related to the risks of data falling into the wrong hands, many participants argued that it is important to be transparent about rights, risks and functionalities of an EPHR to (new) users. According to P9, it is essential to provide an explanation about data privacy in both an accessible language for less informed users, as well as detailed information for individuals with pre-existing knowledge of data protection and privacy. P11 stressed that an EPHR should be promoted as a digital copy of your medical record, a way to be taken seriously by an HCP, not as a ‘miracle cure’ to healthcare access.

Some participants mentioned that people might not be aware of the impact of their medical information being ‘online’. This will allow people to make a critical decision about using an EPHR. As illustrated by P6:

“The context awareness and understanding of what I am actually doing when sharing data is different online, it is more difficult. So, I would say that something like that, […] apart from the actual data itself, should perhaps be more transparent or show more of what is happening.” - P6

Finally, to increase transparency some participants mentioned the importance of specifying the data controller, covered by the GDPR, defined in Article 4(7) as “the natural or legal person, public authority, agency or other body which, alone or jointly with others, determines the purposes and means of the processing of personal data”. This concept and the responsibilities are further elaborated in Article 24 GDPR. P8 noted that when an external party is the data controller in the context of an EPHR, the responsibility for what happens to the data shifts towards this external party. This is important for UDMs or asylum seekers, as it should give them a clear point of contact and accountability. Therefore, P9 stated that it is essential to have clear communication and clarity about who the data controller is.

“Well, I think it is self-evident that data ownership—this sense of ownership over data—forms a very important foundation of the GDPR. This includes privacy law, data protection law, and the right of access to data, as well as the rights individuals have regarding what others do with their data. It also concerns the rights that people, as users of this platform, have in relation to the platform provider.” - P9

##### 3.3.3.3 Educate on functionalities of an EPHRs and people's rights

To mitigate risks of unauthorised access to data in EPHRs, participants frequently emphasised the need to educate both users, healthcare professionals and some mentioned also government agencies.

“And you really do have to educate people on both sides, then of course you have to educate the person concerned, right? They say, don't give your details to so-and-so, but also to anyone else who might ask for them. Remember that you're not allowed to ask.” - P4

It was mentioned by participants that users should understand EPHR functionalities, their rights (e.g., privacy and access to medical records), and their responsibilities, using culturally sensitive and accessible materials. This latter point is important since the concept of privacy could vary among cultures. Participants noted that limited awareness of privacy rights may increase vulnerability. Some participants mentioned that in countries where human rights are often violated, privacy may be perceived not as a fundamental right, but rather as a privilege they often did not have and were therefore less concerned about.

Participants also recommended that HCPs should also be educated about the rights of UDMs and asylum seekers and their professional responsibilities, as participants warned against shifting the responsibility for medical data onto users instead of HCPs. As illustrated by P5:

“But the problem isn't my client, the problem is the professionals, and I want to put it as bluntly as possible. And just let's say, creating a kind of false world and then blaming my clients for it is not right, that's not right, that's where the problem lies.” - P5

##### 3.3.3.4 Make the platform inclusive and involve end-users

Regarding the inclusion of people who might have experienced barriers to using EPHRs such as those with low literacy, several participants raised the importance of making the platform accessible for individuals with a vulnerable health status, low digital skills, or language barriers. Moreover, P12 and P11 raised the importance of involving end-users in the design and implementation of an EPHR.

“I think it could be interesting […] to see whether you could speak with someone who is undocumented or an asylum seeker and who has worked as a physician in their country of origin. […] To see what you could learn from that — also in terms of insight into what people might expect. Some things may be culturally very normal, while we might think, “Huh, that's surprising.”” - P12

## 4 Discussion

This exploratory qualitative socio-legal study described digital health risks for populations living in vulnerable circumstances using the use-case of EPHRs for UDMs and asylum seekers, combining doctrinal legal analysis with expert interviews conducted in the Netherlands. First, it examined the legal frameworks governing the right to health, access to medical records, and (medical) data protection relevant to EPHR adoption, alongside practical barriers to their implementation. This study shows that despite international, European, and Dutch legal guarantees, asylum seekers and UDMs are often unable to fully exercise these rights, due to limited awareness of their rights among these target groups and HCPs, their vulnerable position, which makes it difficult to refuse authority figures, and limited monitoring and sanctioning of right violations.

Second, this study identified key opportunities, barriers, and risks associated with EPHR implementation, including recommendations, which may be relevant to other digital health technologies. Benefits of EPHRs included improved healthcare access and efficiency, and patient empowerment. However, risks were also raised regarding privacy and data protection. Participants identified ‘data falling into the wrong hands’, unauthorised third-party or state access to medical data in an EPHR, as a key risk. Participants feared that data could potentially be used for migration decisions in the future or large scale data collection could be used for automated decision-making systems. Recommendations included the development of a highly secure, privacy-by-design EPHR, transparent communication regarding risks and responsibilities, targeted education for users, HCPs, and government agencies, and the active involvement of end-users in the design. These recommendations are discussed below in the context of objective 1.

Before discussing the recommendations, it is important to further highlight the vulnerabilities of the target groups in relation to this study. Among others, asylum seekers are subject to extensive (personal) data collection (39), while being dependent on authorities for a legal status, accommodation and access to services, which makes it difficult to refuse authorities' requests (40). UDMs may avoid disclosing personal information or accessing healthcare due to concerns about identification, immigration enforcement or deportation, which are recognised barriers to healthcare access among UDMs (41). These concerns are heightened by the European Union's Pact on Migration and Asylum (42), which is applicable as of June 2026. This set of regulations will further expand the collection, interoperability and exchange of personal- and biometric data and facilitates its use for migration control purposes, including faster asylum decision-making, and surveillance, identification, and return management of UDMs in the EU (43, 44). Against this background, the right to non-discrimination in digital health (45) is particularly relevant, as all individuals, including asylum seekers and UDMs, should be able to benefit from digital health technologies such as EPHRs on an equal basis, gaining the improved healthcare access, efficiency, empowerment and control that participants and the literature associate with them (12, 13, 18, 46).

The first recommendation strongly recommended in this study is developing a highly secure EPHR, emphasising privacy-by-design principles. This stems from concerns about the unauthorised access to personal data by third parties or authorities. While some participants noted that data in EPHRs can be used in the advantage of the user in asylum decisions, others feared that medical data could be used against individuals through several routes. The first route, unauthorised access by state authorities or other third parties, is associated with authorities pressuring individuals to provide access to their mobile devices and is closely linked to the described practices of smartphone screening in this study. A practice that often lacks a transparency and a clear legal basis (47). Similar, power imbalances have been described in this study, in line with research that shows that an individual's reluctance to hand over a smartphone to border police raised suspicion and led to additional scrutiny (48). The issue of smartphone screening has also been addressed by the Dutch Council of State (49, 50). The Council stressed that phone searches during asylum procedures constitute an extensive insight into private life and cannot be carried out without a sufficient legal basis. The Dutch Aliens Act provides no such basis. Instead, asylum seekers in the Netherlands were asked to consent to smartphone screening. However, the Dutch Inspectorate of Justice and Security, concluded that requesting consent is inappropriate given the unequal relationship between citizens and the state and asylum seekers’ inadequate information about the screening's purpose, process, and consequences of refusal (51). Despite this, political efforts to establish a legal basis for smartphone searches in asylum procedures have continued and legislative reform is currently being prepared (51).

The second route to unauthorised access, technical breaches, is also recognised in the literature (21, 22, 45). Sun et al. (21) reported that health-sector data breaches are common and can result from malware, cyberattacks, or intentional disclosure by staff, all violating individuals’ right to privacy. Although no specific data on asylum seekers and UDMs has been found, the US Healthcare Data Breach Statistics show a steady upward trend in affected individuals each year, underscoring the need for robust data protection (52).

The third route is unauthorised access via the asylum seekers and UDMs themselves. This underscores that technical safeguards alone are insufficient: users also need to understand the implications of EPHR use and how to use it securely, including how to make informed decisions about their data. This point is also described by Feeney et al. (25), stressing informed consent and user education as essential additional safeguards.

Nonetheless, none of our participants pointed to concrete cases where unlawfully obtained medical data negatively influenced an asylum decision, though several described a “gut feeling” that misuse could occur, particularly if health data were stored on personal devices and because of political uncertanties. This concern seems not merely hypothetical: the WHO Regional Office for Europe (53) reports that health records have at times been used for migration-related decisions elsewhere, and in the U.S., a federal judge blocked an attempt by Medicare and Medicaid Services to share patient data with the Department of Homeland Security for immigration enforcement purposes (54).

These notions highlight the need for additional safeguards, with a strong focus on securing users’ informed consent and understanding (25). Using closed cloud systems with restricted access, strict data minimisation and encryption were recommended, in line with previous research (12, 17, 55). Given the use of phone-screening software, this study also showed the importance of not storing medical data locally on phones, where it may be accessible to such software, but rather in secure, closed cloud-based systems.

Transparency regarding EPHRs' functionalities, risks, and responsibilities emerged as a second recommendation. Participants noted that users may underestimate the value and risks of storing medical data digitally and often share information without fully understanding potential consequences. Clear, simple communication about data risks and safe data practices was therefore considered essential, in line with findings by Namara et al. (56) who argue that easy-to-understand transparent communication about privacy policies could increase comprehension of privacy rights and practices. However, as users may still bypass privacy information or security measures often due to a lack of understanding about the risks involved, transparency alone is insufficient (57). It is recommended to communicate the risks of EPHR usage in a clear and accessible language for users, and in-depth and fact-based for users and lawyers familiar with data security.

Within this context, it is crucial to clearly define and actively communicate who the data controller is regarding EPHRs, as required by the GDPR. Clearly assigning this responsibility enables individuals to effectively exercise their GDPR rights, including the rights relating to information and access, rectification and ensuring the rights to object and to avoid automated individual decision-making. Moreover, it reinforces compliance with the controller's obligations under Articles 24, 25, 32 and 35 of the GDPR, such as implementing appropriate safeguards and conducting Data Protection Impact Assessments (DPIAs), which are particularly important given the sensitive nature of medical data.

Moreover, this study recommended educating users on the secure use of EPHRs, also in line with findings of Tensen et al. (13). Furthermore, research by May (58) reported that educating users can reduce security incidents and foster more responsible use of digital tools. Especially as the study of Tensen et al. (13) and UNHCR underscore the need for increased digital literacy and user education on data protection, given that individuals may lack awareness of the value of their health data (59). For example, a required tutorial on safe data handling practices could be considered before creating an account. Such education must be culturally sensitive and tailored to users’ diverse backgrounds, given persistent misunderstandings and biases within healthcare settings (60). Moreover, educating users on their rights is equally crucial, as many are not aware of their rights to healthcare access, health data access, or data privacy. This finding is in line with research of Teunissen et al. (61), and additionally, it seems that some migrants avoid care due to fears of legal consequences (62). Education can empower them to navigate situations in which an authority or third-party requests access to their health data, or when they wish to obtain a copy of their own medical records.

Our study also underscores the need for education on both the professional and governmental side. HCPs must understand their legal obligations regarding access to healthcare and medical records, a gap also noted in the literature (62). If HCPs share medical information with authorities despite confidentiality obligations, fear of exposure could deter individuals from seeking treatment altogether, with serious public health consequences (63). Furthermore, participants noted gaps in GDPR enforcement and compliance. Although the Dutch Data Protection Authority has imposed fines on public and health sector organisations, its enforcement approach remains only partly transparent (64). Raising awareness within public institutions on handling personal and medical data is therefore essential.

The final recommendation was to make EPHRs inclusive and involve end-users in all stages of development and design, a practice widely supported in the literature (17, 65, 66). Moreover, the WHO (67) notes that community empowerment can be strengthened through inclusive communication approaches that foster increased knowledge and awareness, and ultimately critical thinking (65). An overview of the recommendations on EPHR development and implementation can be found below in Table 3.

**Table 3:** Recommendations on EPHR implementation and development.

Target group	Recommendations
App developers and EPHR implementors	• Develop an EPHR based on privacy-by-design principles, and ensure that the key to data access lies with users. • Do not store medical data on personal mobile devices, instead use encrypted cloud systems. • Clearly define who the data controller is to ensure that responsibilities, as set out in the GDPR, are fulfilled and communicated, providing a reliable point of contact regarding the use and protection of data. • Ensure transparency about functionalities, risks, and responsibilities within an EPHR, allowing users to make informed decisions about account creation and deletion. • Communicate the risks of EPHR usage in clear and accessible language for users, and in-depth fact-based language for users and lawyers familiar with data security. • Educate users about privacy and data protection rights, including how to exercise these rights when authority figures request access to medical information, for example via a mandatory tutorial on safe data handling practices before EPHR account creation. • Provide training for healthcare professionals on rights related to healthcare access for UDMs and asylum seekers, access to health information, and (medical) data privacy. • Raise awareness among institutions and government agencies about the (in)appropriate handling of personal and medical data of UDMs and asylum seekers for migration purposes. • Design systems to be sustainable and accessible for people with limited digital literacy or with a lack of digital means such as internet access. • Involve end-users throughout all stages of design and implementation, ensuring the EPHR is co-created with the intended users.
Policy makers	• Policymakers should prioritise the proper enforcement of existing healthcare and data protection frameworks, including ensuring that GDPR standards are properly interpreted in context of EPHR systems, while taking into account special vulnerabilities of populations with precarious legal statuses. • The data controller concept should be clearly defined and established to avoid accountability gaps and enable the effective exercise of data protection rights. This also includes enforcing compliance with the controller's obligations with regard to transparency, security safeguards, and the systematic use of Data Protection Impact Assessments for systems processing the medical data of vulnerable populations. • Policymakers should take account that consent obtained from UDM and asylum seekers might not reflect actual autonomy, as their dependent position could affect the voluntary and informed nature of their consent.

## 5 Strengths and limitations

This study has several strengths. First, it was conducted by a multidisciplinary team of legal scholars and global health experts, enabling an analysis that bridges legal frameworks, healthcare, and societal perspectives, and provides insight into how laws are applied and experienced in practice from a socio-legal angle. Second, after analysing the interview findings, several experts were consulted to ensure the legal frameworks were comprehensively interpreted. Moreover, while this study is specifically focused on the Netherlands as a use-case, results might also be applicable to other European countries. This study aims to inform policymakers of these gaps between legal frameworks and their practical implementation, and to highlight the need for strengthened enforcement, monitoring, and rights awareness as essential conditions for the responsible and safe implementation of EPHRs for these populations.

Several limitations should be noted. First, the sensitive and politicised nature of migration may have influenced participants’ responses, consciously or unconsciously. Second, this study was based solely on expert interviews and did not include the perspectives of asylum seekers and/or UDMs, and therefore does not provide direct evidence from an UDM or asylum seeker's perspective. This latter point is an important direction for future research. Moreover, as this study chose to focus on both UDMs and asylum seekers, participants did not always specify whether their observations applied to asylum seekers, UDMs, or both, and sometimes used the broader term “migrants”. Future research should more clearly distinguish between these groups.

Third, some experts were unable to participate due to time constraints, potentially limiting practical insights; however, a diverse range of participants was included to mitigate this. Fourth, although WHO reports indicate that medical records have been used for migration-related purposes in parts of Europe, participants in our study were not aware of any concrete examples of such practices. Further research should therefore examine concrete cases in which medical data have been used against individuals and identify individuals willing to share their experiences of data breaches, to support more robust risk-mitigation recommendations. Finally, using both the HRBAD and the OHCHR Human Rights Indicators Framework proved valuable for linking a human-rights based approach to data principles to legal analysis and practice. However, as these frameworks had not previously been combined in research, their integration proved conceptually challenging. Therefore, this lens was applied only during data collection and analysis, rather than as a coherent framework for presenting and discussing the findings. Moreover, as the HRBAD framework (68) is not specifically developed for (medical) data access and protection for individuals, some principles such as ‘transparency’ and ‘data disaggregation’ were not used in this research. To the best of the researcher's knowledge, no framework exists that contains a human rights-based approach to personal (medical) data access and protection. Therefore, it is recommended to explore the development of such a framework.

## 6 Reflexivity

The research team acknowledged their role in shaping the study's focus and interpretation. All authors are part of a multidisciplinary *Digital Health for All* research group, where EPHRs are a central topic, and identified the legal perspective on EPHR development and implementation for UDMs as an under-researched area. P.T. (PhD student and project leader) and Dr. M.D. S.V., both Global Health researchers with long-standing practical and academic experience working with UDMs and EPHRs, shaped the research objectives and methodology. This close involvement may have also introduced certain assumptions regarding the feasibility and desirability of EPHR implementation. Global Health researchers Prof. C.A. (migration and health) and Dr. E.B. (implementation research) contributed a more distanced, analytical perspective, critically challenging underlying assumptions of the other researchers. Interviews with legal experts were conducted by J.M., a master's student in International Health, supported by G.T., a researcher in information law. G.T.'s expertise in legal doctrinal methodologies informed the legal perspective of this study. Her primary expertise lies in Information Law, and Health Law and Migration Law are not her primary domains of expertise. Therefore, additional experts in health and migration law were consulted to review all health-related legal frameworks, ensuring that they reflected the most accurate and up-to-date legal standards.

## 7 Conclusion

This article raises an important dilemma in digital health: whether EPHRs could improve continuity of care for UDMs and asylum seekers, without creating new risks of exclusion, forced disclosure, data misuse, and surveillance of medical data for migration purposes. While UDMs and asylum seekers have the legal rights to healthcare, access to medical records, privacy and protection of medical data, guaranteed under international law, European regulation such as the GDPR, and national Dutch legislation, both enforcement and awareness of these rights remain insufficient. Equal legal protection for these rights is essential: failure to do so risks systemic discrimination and may further heighten the vulnerability of this group. Moreover, if EPHRs are made technically secure through privacy-by-design principles, the question remains whether people in legally precarious positions can meaningfully control, refuse, access, understand, and protect their own health data. At the same time, withholding access to digital health technologies risks excluding these populations further, so the expanding possibilities of digital health must be carefully balanced against such risks. Therefore, guaranteed software safety protecting medical data, transparent, accessible communication about its functioning and risks; education of UDMs, asylum seekers on their rights and secure EPHR usage, education of HCPs, and government agencies, backed by active sanctions for violations; and an inclusive design process involving end-users throughout are recommended. Future research should explore the perspectives of UDMs and asylum seekers themselves, identifying potential experiences of medical data breaches.

## Data Availability

The raw data generated during this study are not publicly available due to the sensitive nature of the data and the risk of participant identification, given the sensitive research topic and small sample size. Requests to access the data should be directed to the corresponding author/s.

## Footnotes

^1^See: Commissie-Klazinga, Arts en vreemdeling. Rapport van de commissie Medische zorg voor (dreigend) uitgeprocedeerde asielzoekers en illegale vreemdelingen, Utrecht, 2007; Derckx & Bloemen, MC 2020/9, p. 18-21 V.L. Derckx & E. Bloemen, ‘Kind zonder verblijfsvergunning heeft ook recht op alle zorg. Belangrijke les uit een schrijnende casus van een asielzoekerskind,’ MC 2020, afl. 9, p. 18-21.)

^2^Formal national requirements for information security. NEN 7510 specifically ensures the safe handling, storage and transfer of personal data.
