# Supplementary material for: Undocumented migrants’ and asylum seekers’ healthcare rights and (medical) data protection: exploring digital health opportunities and risks through the use-case of an electronic personal health record for undocumented migrants and asylum seekers in the Netherlands
Source: Front Digit Health. 2026 Sep 17;8:1876248. doi: 10.3389/fdgth.2026.1876248 (PMC13627290; doi:10.3389/fdgth.2026.1876248)
Supplement: Supplementary file 1 [file Datasheet1.pdf]

Supplementary 1. List of supporting legal references

Box 1: Definition of undocumented migrants and asylum seekers

1951 UN Convention Relating to the Status of Refugees

Convention Relating to the Status of Refugees, adopted 28 July 1951, entered into force 22 April 1954, 189 UNTS 137, Refugee Convention.

Asylum Procedures Directive

Directive 2013/32/EU of the European Parliament and of the Council of 26 June 2013 on common procedures for granting and withdrawing international protection (recast) 2013 (OJ L).

Dutch Aliens Act

Wet van 23 november 2000 tot algehele herziening van de Vreemdelingenwet (Vreemdelingenwet 2000), Stb. 2000, 495.

Qualification Directive

Directive 2011/95/EU of the European Parliament and of the Council of 13 December 2011 on standards for the qualification of third-country nationals or stateless persons as beneficiaries of international protection, for a uniform status for refugees or for persons eligible for subsidiary protection, and for the content of the protection granted (recast) 2011 (OJ L337/9).

Reception Conditions Directive

Directive 2013/33/EU of the European Parliament and of the Council of 26 June 2013 laying down standards for the reception of applicants for international protection (recast) 2013 (OJ L180/96).

Return Directive

Directive 2008/115/EC of the European Parliament and of the Council of 16 December 2008 on common standards and procedures in Member States for returning illegally staying third-country nationals 2008 (OJ L348/98).

Universal Declaration of Human Rights

Universal Declaration of Human Rights (1948) (Proclaimed by the General Assembly, resolution 217 A (III), A/RES/3/217 A, 10 December 1948).

Box 2: Definitions of the right to healthcare

CEDAW

Convention on the Elimination of All Forms of Discrimination against Women (adopted 18 December 1979, entered into force 3 September 1981) 1249 UNTS 13.

Charter

Charter of Fundamental Rights of the European Union [2016] OJ C202/389.

COA Act

Wet van 19 mei 1994, houdende regels betreffende de instelling van een zelfstandig bestuursorgaan, belast met de materiële en immateriële opvang van asielzoekers (Wet Centraal Orgaan opvang asielzoekers), Stb. 1994, 422.

CRC

Convention on the Rights of the Child (adopted 20 November 1989, entered into force 2 September 1990) 1577 UNTS 3.

CRPD

Convention on the Rights of Persons with Disabilities (adopted 13 December 2006, entered into force 3 May 2008) 2515 UNTS 3.

Dutch Aliens Act

Wet van 23 november 2000 tot algehele herziening van de Vreemdelingenwet (Vreemdelingenwet 2000), Stb. 2000, 495.

Dutch Asylum Seekers Provisions Regulation.

Regeling verstrekkingen asielzoekers en andere categorieën vreemdelingen 2005, Stcrt. 2005, 24.

Dutch Constitution

Grondwet voor het Koninkrijk der Nederlanden, Stb. 1983, 70.

Dutch Health Insurance Act

Wet van 16 juni 2005, houdende regeling van een sociale verzekering voor geneeskundige zorg ten behoeve van de gehele bevolking (Zorgverzekeringswet), Stb. 2005, 358.

European Social Charter

European Social Charter (Revised) (adopted 3 May 1996, entered into force 1 July 1999) ETS 163.

ICESCR

International Covenant on Economic, Social and Cultural Rights (adopted 16 December 1966, entered into force 3 January 1976) 993 UNTS 3.

Reception Conditions Directive

Directive 2013/33/EU of the European Parliament and of the Council of 26 June 2013 laying down standards for the reception of applicants for international protection (recast) 2013 (OJ L).

TFEU

Consolidated version of the Treaty on the Functioning of the European Union [2016] OJ C202/47.

Box 3: Right to access personal health data

2024 KNMG Guideline on Medical Data Management

KNMG, *Omgaan met medische gegevens* (KNMG-richtlijn, 2024).

Dutch Civil Code

Burgerlijk Wetboek, BWBR0002656.

GDPR

Regulation (EU) 2016/679 of the European Parliament and of the Council of 27 April 2016 on the protection of natural persons with regard to the processing of personal data and on the free movement of such data, and repealing Directive 95/46/EC (General Data Protection Regulation) (Text with EEA relevance) 2016 (OJ L).

Wabvpz – Act on Additional Provisions for the Processing of Personal Data in Healthcare

Wet aanvullende bepalingen verwerking persoonsgegevens in de zorg, BWBR0023864.

WGBO - Medical Treatment Contracts Act

Wet van 17 november 1994 tot wijziging van het Burgerlijk Wetboek en enige andere wetten in verband met de opneming van bepalingen omtrent de overeenkomst tot het verrichten van handelingen op het gebied van de geneeskunst, Stb. 1994, 837.

Box 4: Description of protection of personal data in GDPR

ECHR - European Convention

Convention for the Protection of Human Rights and Fundamental Freedoms (European Convention on Human Rights, as amended) (ECHR).

Charter

Charter of Fundamental Rights of the European Union [2012] OJ C326/39.

GDPR

Regulation (EU) 2016/679 of the European Parliament and of the Council of 27 April 2016 on the protection of natural persons with regard to the processing of personal data and on the free movement of such data, and repealing Directive 95/46/EC (General Data Protection Regulation) (Text with EEA relevance) 2016 (OJ L).
