# Supplementary material for: Undocumented migrants’ and asylum seekers’ healthcare rights and (medical) data protection: exploring digital health opportunities and risks through the use-case of an electronic personal health record for undocumented migrants and asylum seekers in the Netherlands
Source: Front Digit Health. 2026 Sep 17;8:1876248. doi: 10.3389/fdgth.2026.1876248 (PMC13627290; doi:10.3389/fdgth.2026.1876248)
Supplement: Supplementary file 2 [file Datasheet2.pdf]

Supplementary 2. List of supporting legal abreviations

National

Dutch Aliens Act 2000 (Vreemdelingenwet 2000)

Dutch Asylum Seekers and Other Categories of Aliens Provisions Regulations (Regeling Verstrekkingen Asielzoekers en Andere Categorieën Vreemdelingen 2005)

COA Act (Wet Centraal Orgaan opvang asielzoekers)

Medical Treatment Contracts Act (Wet op de Geneeskundige Behandelingsovereenkomst (WGBO)

Dutch Civil Code (Burgerlijk Wetboek (BW))

Act on Additional Provisions for the Processing of Personal Data in Healthcare (Wet aanvullende bepalingen verwerking persoonsgegevens in de zorg (Wabvpz))

Individual Healthcare Professions Act (Wet BIG)

Dutch Constitution (Grondwet)

Health Insurance Act (Zorgverzekeringswet)

Regulation on Medical Care for Asylum Seekers (Regeling Medischezorg Asielzoekers (RMA))

European

ECHR - European Convention for the Protection of Human Rights and Fundamental Freedoms (adopted 4 November 1950, entered into force 3 September 1953) ETS No 5

Charter - Charter of Fundamental Rights of the European Union [2012] OJ C 326/391

European Social Charter (revised) (adopted 3 May 1996, entered into force 1 July 1999) ETS No 163

Qualification Directive 2011/95/EU (recast) - Directive 2011/95/EU of the European Parliament and of the Council of 13 December 2011 on standards for the qualification of third-country nationals or stateless persons as beneficiaries of international protection, for a uniform status for refugees or for persons eligible for subsidiary protection, and for the content of the protection granted (recast) [2011] OJ L 337/9

Asylum Procedures Directive 2013/32/EU (recast) - Directive 2013/32/EU of the European Parliament and of the Council of 26 June 2013 on common procedures for granting and withdrawing international protection (recast) [2013] OJ L 180/60

Reception Conditions Directive 2013/33/EU (recast) - Directive 2013/33/EU of the European Parliament and of the Council of 26 June 2013 laying down standards for the reception of applicants for international protection (recast) [2013] OJ L 180/96.

TFEU - Treaty on the Functioning of the European Union (TFEU) [2012] OJ C 326/47

GDPR - Regulation (EU) 2016/679 of the European Parliament and of the Council of 27 April 2016 on the protection of natural persons with regard to the processing of personal data and on the free movement of such data, and repealing Directive 95/46/EC (General Data Protection Regulation) [2016] OJ L 119/1

Directive 2008/115/EC of the European Parliament and of the Council of 16 December 2008 on common standards and procedures in Member States for returning illegally staying third-country nationals [2008] OJ L 348/98.

EU documents / policy

European Commission, Communication on a New Pact on Migration and Asylum COM(2020) 609 final

European Commission, Proposal for a Regulation on the European Health Data Space COM(2022) 197 final

International

UDHR - Universal Declaration of Human Rights (adopted 10 December 1948) UNGA Res 217 A (III)

Convention Relating to the Status of Refugees (adopted 28 July 1951, entered into force 22 April 1954) 189 UNTS 137

ICESCR - International Covenant on Economic, Social and Cultural Rights (adopted 16 December 1966, entered into force 3 January 1976) 993 UNTS 3
