# Supplementary material for: Undocumented migrants’ and asylum seekers’ healthcare rights and (medical) data protection: exploring digital health opportunities and risks through the use-case of an electronic personal health record for undocumented migrants and asylum seekers in the Netherlands
Source: Front Digit Health. 2026 Sep 17;8:1876248. doi: 10.3389/fdgth.2026.1876248 (PMC13627290; doi:10.3389/fdgth.2026.1876248)
Supplement: Supplementary file 4 [file Datasheet4.pdf]

Supplementary 4. Interview guide (English)

[organization] is working on a project called HealthEmove, an Electronic Personal Health Record (EPHR) designed for "people on the move"—in this case, undocumented migrants and asylum seekers. To help protect their rights, we are investigating how access, ownership, and protection of their health data can be safeguarded. This involves analysing the legal frameworks related to health data and these vulnerable populations.

An EPHR is a digital tool that allows individuals to manage, access, and share their own health information with new healthcare providers. Unlike Electronic Health Records (EHRs), which are managed by healthcare institutions, EPHRs are managed by the users themselves. This offers individuals more control over their medical data and promotes autonomy in healthcare.

EPHRs typically include information such as medical history, treatment plans, medications, allergies, and vaccinations. HealthEmove is built on an existing Dutch personal health environment (PGO) called Patient Knows Best (PKB), implemented through a white-label construction. This allows the platform to be used free of charge. PKB complies with the Dutch MedMij standards, which were developed to ensure safe and interoperable data exchange between patients and healthcare providers.

This will be an open conversation. I will start with some general introductory questions to understand your perspective, and then we will move on to various aspects of the legal framework and the implementation of digital health records. The interview will take approximately 45 minutes. Your responses will be fully anonymised and securely stored. The recording will be used for analysis purposes only and will help ensure the information is processed accurately.

HealthEmove is a type of personal health environment (PGO) that differs from other systems in that it does not use the Dutch citizen service number (BSN) to link personal data. Instead, it works with unstructured data. This offers a different perspective on how health information can be managed and shared—especially for vulnerable groups such as undocumented migrants and asylum seekers.

The data is stored on a Google Cloud server located in [location]. Technically, this is as secure as systems used by hospitals, although it may still raise concerns. Information can be added either by the user themselves or by a healthcare provider who has been granted access and also holds an account.

<u>Section 1 - Introduction</u>

Can you please introduce yourself?

1. What are your thoughts on the concept of an EPHR as explained?
    1. Do you consider such a system safe enough in theory and practice?
2. Have you encountered situations in your work where clients' medical data played a role in asylum or immigration procedures?

<u>Section 2 - Structural indicators</u>

Which legal frameworks are most relevant for protecting the medical data of UDMs or asylum seekers in the Netherlands?

4. Does the GDPR provide sufficient safeguards in this context?

5. What legal rights do UDMs and asylum seekers have regarding accessing their own medical records?

    1. Is there a difference in the legal rights of UDMs and asylum seekers regarding access to their medical records?
    2. What (legal) factors might lead to a refusal of access to these records?
    3. What potential costs are associated with accessing their medical records?

6. Under what circumstances are healthcare providers or other entities allowed to share the medical information of UDMs and asylum seekers with third parties, such as government agencies, law enforcement, other healthcare providers, attorneys, or relevant organizations?

    1. What legal justification exists for sharing this data?

7. Under what conditions may third parties (e.g. police or IND) access EPHRs?

    1. What actions can they take with this information?

8. How do these regulations differ from civilians with legal citizenship?

<u>Section 3 - Process indicators</u>

10. Can UDMs or asylum seekers effectively exercise their rights in practice?

11. What legal or institutional barriers hinder the enforcement of those rights?

12. What are the main risks when vulnerable groups share medical data through an EPHR?

13. What safeguards are needed to prevent misuse of personal data?

14. How are rights and responsibilities communicated to users in practice?

15. What happens if something goes wrong? Are there procedures for complaints or redress?

16. Are there effective enforcement mechanisms in place? Who oversees this?

17. Have you witnessed cases where authorities accessed clients' digital or medical data?

    1. How was this justified or challenged legally?

18. In your view, how can a digital system like an EPHR be designed to support these users' rights more effectively?

<u>Section 4 - Outcome indicators</u>

19. What are the legal and policy challenges to ensuring rights to access, ownership, and control over EPHRs for vulnerable groups?

20. Do you have concerns or recommendations regarding the digitalisation of health data in the context of asylum procedures?

21. What would be your main legal or institutional recommendations to improve protections?

22. What are the implications of combining structured and unstructured data (e.g. screenshots, photos)?

    1. Does this still fall within a medical context, or does the legal framework shift?

23. What are the privacy implications of storing such data, especially without using a BSN?

    1. Are there viable alternatives to BSN-based identification, and what would be the impact?

24. The software is partly developed in the UK, but hosted in Amsterdam. What are the legal implications for cross-border data transfer under the GDPR?

25. Any final thoughts on data protection, legal rights, or system design that you believe are important to mention?
